# The clinical effectiveness and cost-effectiveness of a ‘stepping into day treatment’ approach versus inpatient treatment as usual for anorexia nervosa in adult specialist eating disorder services (DAISIES trial): a study protocol of a randomised controlled multi-centre open-label parallel group non-inferiority trial

**DOI:** 10.1186/s13063-022-06386-7

**Published:** 2022-06-16

**Authors:** Madeleine Irish, Bethan Dalton, Laura Potts, Catherine McCombie, James Shearer, Katie Au, Nikola Kern, Sam Clark-Stone, Frances Connan, A. Louise Johnston, Stanimira Lazarova, Shiona Macdonald, Ciarán Newell, Tayeem Pathan, Jackie Wales, Rebecca Cashmore, Sandra Marshall, Jon Arcelus, Paul Robinson, Hubertus Himmerich, Vanessa C. Lawrence, Janet Treasure, Sarah Byford, Sabine Landau, Ulrike Schmidt

**Affiliations:** 1 Section of Eating Disorders, Department of Psychological Medicine, Institute of Psychiatry, Psychology & Neuroscience, King’s College London, De Crespigny Park, London, SE5 8AF UK; 2 Department of Biostatistics and Health Informatics, Institute of Psychiatry, Psychology & Neuroscience, King’s College London, De Crespigny Park, London, SE5 8AF UK; 3 Department of Health Service and Population Research, Institute of Psychiatry, Psychology & Neuroscience, King’s College London, De Crespigny Park, London, SE5 8AF UK; 4 South London and Maudsley NHS Foundation Trust, London, UK; 5 Gloucestershire Health and Care NHS Foundation Trust, Gloucester, UK; 6 Central and North West London NHS Foundation Trust, London, UK; 7 NHS Grampian, Aberdeen, UK; 8 South West London and St George’s Mental Health NHS Trust, London, UK; 9 NHS Dumfries and Galloway, Dumfries, UK; 10 Dorset HealthCare University NHS Foundation Trust, Poole, UK; 11 Surrey and Borders Partnership NHS Foundation Trust, Leatherhead, UK; 12 Leicestershire Adult Eating Disorders Service, Leicestershire Partnership NHS Trust, Bennion Centre, Glenfield Hospital, Leicester, UK; 13 Institute of Mental Health, University of Nottingham, Jubilee Campus, Triumph Road, Nottingham, NG7 2TU UK; 14 Division of Medicine, University College London, 5 University Street, London, WC1E, 6JF UK

**Keywords:** Anorexia nervosa, Inpatient treatment, Day patient treatment, Partial hospitalisation, Intensive treatment, Stepped care

## Abstract

**Background:**

Anorexia nervosa (AN) is a serious and disabling mental disorder with a high disease burden. In a proportion of cases, intensive hospital-based treatments, i.e. inpatient or day patient treatment, are required, with day patient treatment often being used as a ‘step-down’ treatment after a period of inpatient treatment. Demand for such treatment approaches has seen a sharp rise. Despite this, the relative merits of these approaches for patients, their families, and the NHS and wider society are relatively unknown. This paper describes the rationale for, and protocol of, a two-arm multi-centre open-label parallel group non-inferiority randomised controlled trial, evaluating the effectiveness and cost-effectiveness of these two intensive treatments for adults with severe AN: inpatient treatment as usual and a stepped care day patient approach (the combination of day patient treatment with the option of initial inpatient treatment for medical stabilisation). The main aim of this trial is to establish whether, in adults with severe AN, a stepped care day patient approach is non-inferior to inpatient treatment as usual in relation to improving body mass index (BMI) at 12 months post-randomisation.

**Methods:**

386 patients with a Diagnostic and Statistical Manual 5th edition diagnosis of severe AN or related disorder, with a BMI of ≤16 kg/m^2^ and in need of intensive treatment will be randomly allocated to either inpatient treatment as usual or a stepped care day patient approach. Patients in both groups will receive treatment until they reach a healthy weight or get as close to this point as possible. Assessments will be conducted at baseline (prior to randomisation), and at 6 and 12 months post-randomisation, with additional monthly symptom monitoring. The primary outcome will be BMI at the 12-month post-randomisation assessment. Other outcomes will include psychosocial adjustment; treatment motivation, expectations and experiences; cost-effectiveness; and carer burden.

**Discussion:**

The results of this study will provide a rigorous evaluation of two intensive treatment approaches which will inform future national and international treatment guidelines and service provision.

**Trial registration:**

ISRCTN ISRCTN10166784. Registered 28 February 2020. ISRCTN is a primary registry of the World Health Organization (WHO) International Clinical Trials Registry Platform (ICTRP) network and includes all items from the WHO Trial Registration Data Set.

## Background

Anorexia nervosa (AN) is a serious mental disorder associated with high levels of mortality and disability, physical and psychological morbidity, and impaired quality of life [1–3]. At a time when admission rates for most other major mental disorders (e.g. schizophrenia, depression, bipolar disorder and dementia) have fallen [4], hospital admission rates for AN have seen a sharp rise. For example, the Oxford Record Linkage Study reported that the age standardised first-recorded admission rate for women (aged 10–44 years) has increased from 2.7 per 100,000 of the female population in 1968–1971 to 6.3 in 2007–2011 and for males, 0 per 100,000 in the 1960s to 0.4 per 100,000 in 2007–2011 [5]. Country-wide data have shown a similar trend [5]. More recently, NHS Digital data have revealed that admissions for eating disorders (EDs) have risen 37% across all age groups over a 2-year period from 2016/17 and 2018/19 [6]. A potential alternative to inpatient treatment for severely unwell patients with AN is day treatment. Often this is used as a step-down treatment after an initial period of inpatient treatment for medical stabilisation [7–9]. However, the relative merits of these two intensive treatment approaches for patients with severe AN, their families, and the NHS and wider society are relatively unknown. Therefore, this study aims to compare the clinical and cost-effectiveness of inpatient treatment as usual (IP-TAU), the current standard care pathway, to a stepped care day treatment approach, which combines intensive day patient treatment with the option for initial inpatient treatment for medical stabilisation and progression to day patient treatment at the earliest opportunity, for adults with severe AN.

Both of these intensive treatment approaches have potential risks and benefits for both the patient and their carers. IP-TAU is widely considered the gold standard for patients with severe AN, as it offers intensive support around feeding and safety monitoring. It may also give families a much-needed respite from looking after their relative. Patients may feel that they are part of a ready-made community of people with similar problems. However, especially if the admission is prolonged, as is common, it may lead to patients becoming institutionalised, passive and disempowered and make it harder for them to translate gains made whilst in hospital into their life in the community. In contrast, a stepped care day treatment approach allows for the delivery of flexible personalised care tailored according to patient risk and progress and for patients to retain better links with their family and friends. It may also help them realise that they need to be actively involved in their own recovery, and by doing so may make them more resilient against relapse and able to cope with any setbacks. Likewise, this approach may also help carers feel more empowered to support the person at home. However, having intensive support only for part of the week may make it harder to achieve substantial weight gain, necessary for recovery. Daily travel may pose practical challenges for patients living at a distance and having to spend evenings and weekends at home may increase feelings of loneliness and isolation. It may also increase burden on families. Ultimately, the risk and benefit ratio will vary from case to case and it is currently unknown whether treatment outcomes from these approaches are comparable.

Few studies have compared the clinical effectiveness of these intensive treatment approaches. We searched systematic reviews of published AN treatment trials [10, 11], and reviews of trials assessing different intensive treatment settings [12–14], including day patient treatment programmes [9, 15–17] and inpatient treatment [10, 18]. We identified only one randomised controlled trial (RCT) comparing inpatient treatment to a stepped care day patient treatment approach in severe AN [19] and a further two studies (*n* = 1 RCT [20] and *n* = 1 case-control study [21]) that compared inpatient to intensive day patient treatment (without the option for stepped care) in severe AN. In these comparisons of day to inpatient treatment, one case-control study (*n* = 26) reported a superiority of inpatient treatment, over day patient treatment [21] and the other small RCT (*n* = 32) found no difference in outcome between the two approaches (reviewed in [18, 20]). More recently, a large (*n* = 172) well-conducted trial in adolescents with first episode AN in Germany found that patients could safely be stepped down to day treatment after a 3-week inpatient admission and that this stepped care approach was non-inferior to inpatient treatment [19]. Furthermore, at a 2-year follow-up, social outcomes were better in stepped care day patient treatment than in the inpatient treatment arm (Herpertz-Dahlmann, personal communication). Through an additional database (PubMed) search, we identified a recent retrospective case-control study comparing outcomes of inpatient and day patient treatment in 34 adolescents with AN [22]. Patients admitted to inpatient treatment were matched with patients admitted to day patient treatment on age, BMI and clinical status. Whilst the two treatment groups did not differ at admission, at discharge, patients who received day patient treatment had greater weight gain and improvement in ED symptoms and psychosocial functioning. Evidence of weight and psychosocial outcomes for inpatient and day patient treatment, and in particular a stepped care approach, is therefore limited.

In addition to patient-specific outcomes (i.e. weight and psychosocial outcomes), the impact on a system level, e.g. the cost-effectiveness of the two approaches, is also an important consideration. AN has one of the highest treatment costs of any psychiatric disorder [23–25]. This is largely due to the high cost of inpatient treatment, which is directly related to illness severity (i.e. body mass index [BMI]), as this affects length of stay [25]. Guarda et al. [26] reported that whilst, in the USA, the average cost per day was $2295 for inpatient treatment and $1567 for day treatment, the average cost per pound in weight gained was $4089 and $7050 respectively, suggesting that inpatient treatment may be a more efficient way of achieving weight recovery. However, this study only focused on cost related to short-term weight outcomes, and longer-term weight and psychological outcomes were not considered. In contrast, the previously described RCT by Herpertz-Dahlmann et al. [19] found that the stepped care day patient treatment approach (i.e. a short inpatient admission followed by day treatment) for adolescents with AN was less costly than inpatient treatment, and the previously described study by Zanna et al. [22] reported no significant difference in costs between inpatient and day patient treatment.

At present, it is unclear how a day patient treatment approach compares to inpatient treatment for an adult population with severe AN within the context of different health care systems. To gauge how inpatient and day treatments are used for adults with AN, we reviewed admission BMIs and length of stay in naturalistic studies of these treatments. For day treatments, based on a narrative review [9] and two further studies [7, 26], we identified 11 uncontrolled studies (*n* = 1010 AN patients) from different countries (USA, Canada, Australia, Germany, Italy, Holland, UK). Across these studies, admission BMIs ranged from 15.7 to 18.7 kg/m^2^. With the exception of one study [26], all others had a mixture of patients who were stepped down from inpatient treatment or admitted from the community. Length of stay ranged from 32 to 182 days, with most programmes offering treatment for 10–16 weeks, and a recent meta-analytic review found a mean length of stay of 86.3 days [13]. For inpatient treatment, 15 adult inpatient cohorts (*n* = 2100) from different countries (USA/Canada, Australia/New Zealand, Germany, Italy, UK) reported mean admission BMIs ranging from 13.9 to 16.3 kg/m^2^ [26–42] and a meta-analysis reported a mean length of stay of 105.6 days across Europe and 49.1 days across USA [13]. A recent audit from the Quality Network for Eating Disorders in the UK, which included data submitted from all adult inpatient eating disorder services in England (*n* = 30) and two in Scotland (out of four adult inpatient eating disorder services), reported the mean length of stay for inpatient treatment as 116 days [43]. Taken together, these data suggest that internationally there is considerable variation in the severity and length of stay across intensive settings for patients with AN and the optimal length of intensive treatments for AN is unknown. Furthermore, it can be concluded that, internationally, day treatment is typically used for patients that do not have severe AN (as defined by BMI). Given this, there is limited evidence for the use of day treatment for our target population of severe AN.

In summary, relatively little is known about the comparative effectiveness, cost and cost-effectiveness of a stepped care day patient approach to inpatient treatment as usual for treating severe AN. If at least a proportion of patients needing intensive treatment could be treated as day patients, or be stepped down into day treatment from initial inpatient treatment, this could have significant cost savings. The proposed trial (the DAISIES study) aims to compare these two intensive treatment approaches in a two-arm multi-centre open-label parallel group non-inferiority RCT in adults with severe AN or related disorders in the National Health Service (NHS) of the UK.

### Study objectives

The specific objectives of the proposed RCT study are to:

1. Establish whether a stepped care day patient approach is non-inferior to inpatient treatment as usual (IP-TAU) in relation to improving BMI at 12 months post-randomisation (primary outcome).
2. Compare the two care pathways in terms of AN symptoms, comorbid symptoms and psychosocial outcomes at different time points (superiority assessment).
3. Establish whether a stepped care approach is cost-effective compared to inpatient treatment as usual (IP-TAU) in terms of quality-adjusted life-years (QALYs) at 12 months post-randomisation.
4. Investigate the experiences of and views on the treatment approaches from the perspective of patients, families and clinicians to provide insight into mechanisms of impact and how context and implementation inform outcomes.

Whilst this study was originally planned prior to the start of the Covid-19 pandemic, adjustments were made to the protocol due to the profound impact of the virus on ED patients [44–46] and on inpatient and day patient services in the UK. These changes will be explained in the relevant sections below.

## Methods and analysis

This study protocol has been written in accordance with the SPIRIT (Standard Protocol Items: Recommendations for Interventional Trials) statement [47].

### Study design

The DAISIES trial is a pragmatic two-arm multi-centre open-label parallel group non-inferiority RCT, rated as highly pragmatic on the PRECIS-2 tool [48]. It compares two intensive treatment approaches within a standard NHS setting: (a) specialist inpatient treatment as usual (IP-TAU) or (b) a stepped care day patient approach. We considered three design options, all for a non-inferiority trial. Firstly, we considered a direct comparison between intensive day patient treatment with inpatient treatment for those who could safely be allocated to day patient treatment. However, this would mean that the study’s target population would be restricted to those patients who could immediately be allocated to day patient treatment. This would exclude large numbers of patients from the study population and would severely reduce generalisability of the trial results. Secondly, we considered modelling our trial on the previously described large well-conducted German trial in adolescents with a first episode of AN [19]. In this trial, all patients initially had a brief (3 weeks) inpatient treatment for medical stabilisation and were then randomly allocated to either continue with inpatient treatment or to step down to day patient treatment. However, adults with AN are more heterogeneous than adolescents in terms of medical risk and thus a fixed-duration initial inpatient stay would ‘over-treat’ some patients and ‘under-treat’ others. Finally, we considered (and decided to use) the more flexible stepped care approach described below, as this allows delivery of personalised care, tailoring intervention according to patient risk and progress.

Patients will be allocated to either treatment approach at a 1:1 ratio. To assess recruitment rates, an internal pilot trial (aiming to recruit 62 patients over 4 months) is included in the study design. If successful, we will continue with the full study which will include 386 adults (including pilot participants) with severe AN or related disorders (e.g. avoidant restrictive food intake disorder [ARFID]), deemed to need intensive treatment. ED symptoms and BMI will be monitored on a monthly basis from baseline assessment (prior to randomisation) to 12 months post-randomisation, and more comprehensive assessments will take place at baseline, and at 6 and 12 months post-randomisation.

A further 24-month follow-up study is planned, as improvements in both nutrition and psychological functioning can continue over a number of years. Whilst for a proportion (~ 60%) of participants, the 24-month assessment will take place during the funded trial period, further funding will be sought to continue with the 24-month assessments. These assessments do not form part of the DAISIES trial and are not covered in this trial protocol.

### Study setting

This study will take place in a number of specialist ED services across the UK. At the time of publication, 10 services were included as sites (see Trial Registration at 10.1186/ISRCTN10166784 for an up-to-date list of participating sites). The study was advertised to all eating disorder services in the UK via the listserves of the Royal College of Psychiatrists’ Eating Disorders Faculty and the British Eating Disorders Society. To participate in the study, study sites were required to have an inpatient and day patient service for adults with AN. In order to include as many day patient services as possible, sites which only had a day patient service (but no inpatient service) were included if the local services used by the site for providing inpatient care provision were also willing to participate in the trial.

### Participants and recruitment

Adults with severe AN or ARFID, with a BMI of 16.0 kg/m^2^ or below, and who are in need of intensive treatment will be recruited from specialist ED inpatient and outpatient services. Our definition of severe AN is in accordance with the World Health Organization’s definition of severe thinness as a BMI < 16 kg/m^2^ [49] and the Diagnostic and Statistical Manual of Mental Disorders (DSM)-5 definition of severe AN [50].

#### Inclusion and exclusion criteria

Inclusion criteria are as follows: (1) adults aged 17 years or above; (2) DSM-5 [50] diagnosis of AN or ARFID; (3) BMI of equal to or less than 16.0 kg/m^2^; (4) in need of intensive treatment because of either rapid weight loss, and/or evidence of system/organ failure/medical instability and/or unsuccessful outpatient treatment, as defined by NHS England [51]; and (5) have mental capacity to give informed consent to participate in the study. Exclusion criteria are as follows: (1) individuals with insufficient knowledge of English to complete study assessments or understand treatment; (2) individuals with severe learning disabilities; (3) individuals with a severe medical or psychiatric (co)morbidity (e.g. psychosis, substance dependence) requiring treatment in its own right; and (4) those living too far away from day patient treatment (and where no alternative arrangements for regular attendance at day patient treatment can be made).

#### Sample size

The sample size calculation is based on a one-sided non-inferiority test at 97.5% confidence. Previous studies suggested a standard deviation for within-group BMI at 12 months of 2.3 kg/m^2^ [19, 38]. Based on Herpertz-Dahlmann et al. [19], we defined our non-inferiority threshold as a 0.75 kg/m^2^ decrease in BMI for stepped care relative to IP-TAU. If there is truly no difference between stepped care and IP-TAU, then 198 participants per trial arm are needed to be 90% sure that the lower limit of a one-sided 97.5% confidence interval (or equivalent a 95% two-sided confidence interval) for the difference will be above the non-inferiority limit of −0.75. To account for the precision gain due to including baseline BMI in the modelling, we applied a deflation factor (0.78, based on a pre-post correlation of 0.47, [38]) to the estimated sample size [52]. We also inflated the requirement to account for 20% dropout at 12 months based on our previous studies [53, 54]. This gave a final sample size requirement of 193 per trial arm (*n* = 386 in total).

### Randomisation

The generation and implementation of the randomisation sequence will be conducted independently from the trial team through an online system provided by King’s Clinical Trials Unit (KCTU). Randomisation will be done at the level of the individual and stratified for known prognostic variables of outcomes (previous inpatient treatment [yes/no], illness duration [≤ or > 3 years] and recruitment centre). Minimisation with a stochastic component will be used to balance these three prognostics variables across trial arms. After the baseline assessment is completed, participant ID and stratification details will be entered into the web-based KCTU system by a member of the research team. Participants will then be randomly allocated on a 1:1 ratio to either (a) IP-TAU or (b) a stepped care day patient approach. A designated unblinded researcher in the trial team will oversee randomisation. This includes receiving the randomisation outcome from the online system and informing the participant’s treating clinical team of the allocated treatment arm. It will be the responsibility of the participant’s treating clinical team to inform the participant of their allocated treatment.

### Blinding

Due to the nature of the intervention, neither participants (patients and carers) nor treating clinicians can be blinded to the treatment allocation and, as such, will be aware of the type of care they are delivering and being offered, respectively. Participants will be strongly encouraged not to disclose their treatment allocation to the researchers at the follow-up assessments. The trial health economists will also be aware of the type of care offered to and received by participants (i.e. unblinded) due to the identifying nature of service use data. There will be one unblinded researcher (who will oversee randomisation as described above) and they will not perform any follow-up data collection, with the exception of data that may cause unblinding (e.g. service use data as measured by the Adult Service Use Schedule). The trial statistician will be blind until the sign off of the Statistical Analysis Plan. The remaining research assessors and the senior trial statistician will be blind to intervention allocation. Blinding success of blinded researchers will be assessed at 6 and 12 months post-randomisation. Blinded researchers (assessors and senior trial statistician) will be unblinded at the end of the study.

### Treatment approaches

#### Inpatient treatment as usual

IP-TAU uses the current standard patient care pathway. The aim of this care pathway is for patients to normalise their eating and reach a healthy weight or get as close to this as possible. Patients admitted to IP-TAU will be treated by a multidisciplinary team (including psychiatrists, psychologists, dieticians, nurses and others) and receive expert refeeding, therapeutic programmes and supervised meals and snacks. There is no concomitant care prohibited during the trial. A proportion may go on to have day patient treatment at the end or be discharged to outpatient treatment, at the discretion of the treating team. This decision will often depend on pressures on beds and to some extent, also on patient preferences. However, every attempt will be made to retain patients in the inpatient arm until they have completed their course of inpatient treatment. If the patient goes on to receive day patient treatment following their inpatient admission at one of the study sites, data on this day treatment (e.g. admission and discharge data, attendance) will be collected until end of follow-up (i.e. 12 months post-randomisation). Any patient, if required, can be re-admitted to inpatient treatment. This will not count towards their completion of IP-TAU.

#### Stepped care day patient approach

This stepped care treatment approach combines intensive day patient treatment with the option of inpatient treatment for medical stabilisation and progression to day patient treatment at the earliest opportunity. The aim of this care pathway is for patients to normalise their eating and reach a healthy weight or get as close to this as possible.

The clinical team will complete an initial decision tool (described below) on all participants when assessing the patient’s eligibility for the study (i.e. prior to randomisation) to help decide on whether the patient ought to start this treatment pathway with inpatient or day patient treatment. Within the stepped care treatment arm, further weekly decision tools will be completed for the duration of intensive treatment (i.e. in- or day patient treatment) with clear rules around patients’ suitability for stepping down into specialist day patient treatment (or stepping up from day patient treatment into inpatient treatment if necessary, in the case of deterioration or relapse). Patients should step down to day patient treatment within 1 month of being at an appropriate level of risk, as detailed below. Day patient treatment will involve a full-time programme covering 4–5 days a week with 2–3 meals per day, multidisciplinary support (including psychiatrists, psychologists, dieticians, nurses and others) and high-quality evidence-based psychological interventions for patients and their carers. There is no concomitant care prohibited during the trial. Patients will return home for weekends and evenings. As a result of the Covid-19 pandemic, a protocol change was made so that day patient treatment can now also be delivered using a blended or hybrid approach, combining both remote (video-conferencing) and physical attendance at day service activities (e.g. supported meals, groups) and psychological therapies. Having this flexibility will allow us to react to the constantly changing situation and continue with the trial if later Covid-19-related local/national lockdowns are enforced resulting in services having to temporarily halt face-to-face treatment. Data will be collected from the clinical teams until end of follow-up (i.e. 12-months post-randomisation) to allow us to fully characterise patients’ treatment. This includes admission and discharge dates for day patient treatment and any inpatient admissions (if required for medical stabilisation), weekly day patient treatment attendance, and also proportion of remote versus physical attendance.

#### Definition of treatment completion

The length of intensive treatment for AN (inpatient or day treatment setting) is not pre-specified in the UK. In this trial, we use two criteria to determine essential and enhanced treatment completion respectively: essential treatment completion is defined as having received at least 8 weeks of intensive treatment (~ 50% of the mean length of stay, [13, 43]) and enhanced treatment completion is defined as having received at least 12 weeks (around 80% of the mean length of stay, [13, 43]) of intensive treatment. For the IP-TAU arm, this definition is straight forward, except that in some cases patients will already be an inpatient at the point of randomisation. In these cases, only the time spent as an inpatient post-randomisation ‘counts’ towards study treatment completion. For the stepped care day treatment arm, the definition is more complex, as some day services start patients on a full-time programme but move to part-time day treatment to facilitate patients gradually returning to other life activities, or operate alternating full-time, part-time regimes. Therefore, whilst essential treatment completion is defined as at least 8 weeks of full-time day treatment, enhanced treatment completion is defined as either 12 weeks of full-time day treatment or 8 weeks of full-time day treatment and 8 weeks of part-time day treatment. For both treatment arms, the allocated intervention may be modified or discontinued if clinically indicated or due to patient request.

#### Decision tool

This is based on a modified version of the Maudsley Medical Risk Assessment tool [55] which uses a traffic light system of quantifying risk. Variables from the evidence-based MARSI MEWS [56, 57] will be added. Our decision tool comprehensively assesses medical risk, using objective indicators of nutritional status (e.g. BMI, weight change), cardiovascular function (e.g. blood pressure, pulse, postural drop), laboratory parameters and other physical risk indicators. In addition, we have added a psychiatric/psychosocial risk category (including, e.g. suicidality, major self-harm, availability of support, safe-guarding concerns, patient/carer concerns). These risk indicators will be combined into a one-page easy-to-use proforma. This decision tool will be completed for all patients who are assessed for study eligibility and also for those participants randomly allocated to the stepped care day treatment approach: a senior clinician will repeat this decision tool on a weekly basis from the start to end of intensive treatment (in- and day patients). This tool will facilitate decision making around the most appropriate setting of treatment for the patient at that time. Patients with any indicators in the red risk category will usually be admitted to or continue inpatient treatment. Patients whose risk indicators fall exclusively or predominantly into the green category (with isolated or borderline amber indicators only) will start or be stepped down into day patient treatment. Patients with several indicators in the amber category will usually be admitted to/stay in inpatient treatment until there are clear signs of improvement. If an inpatient has remained on amber for ≥ 4 weeks, their scores will be discussed with the risk reference committee, to ensure that there is consensus.

### Procedure

**Fig. 1:**
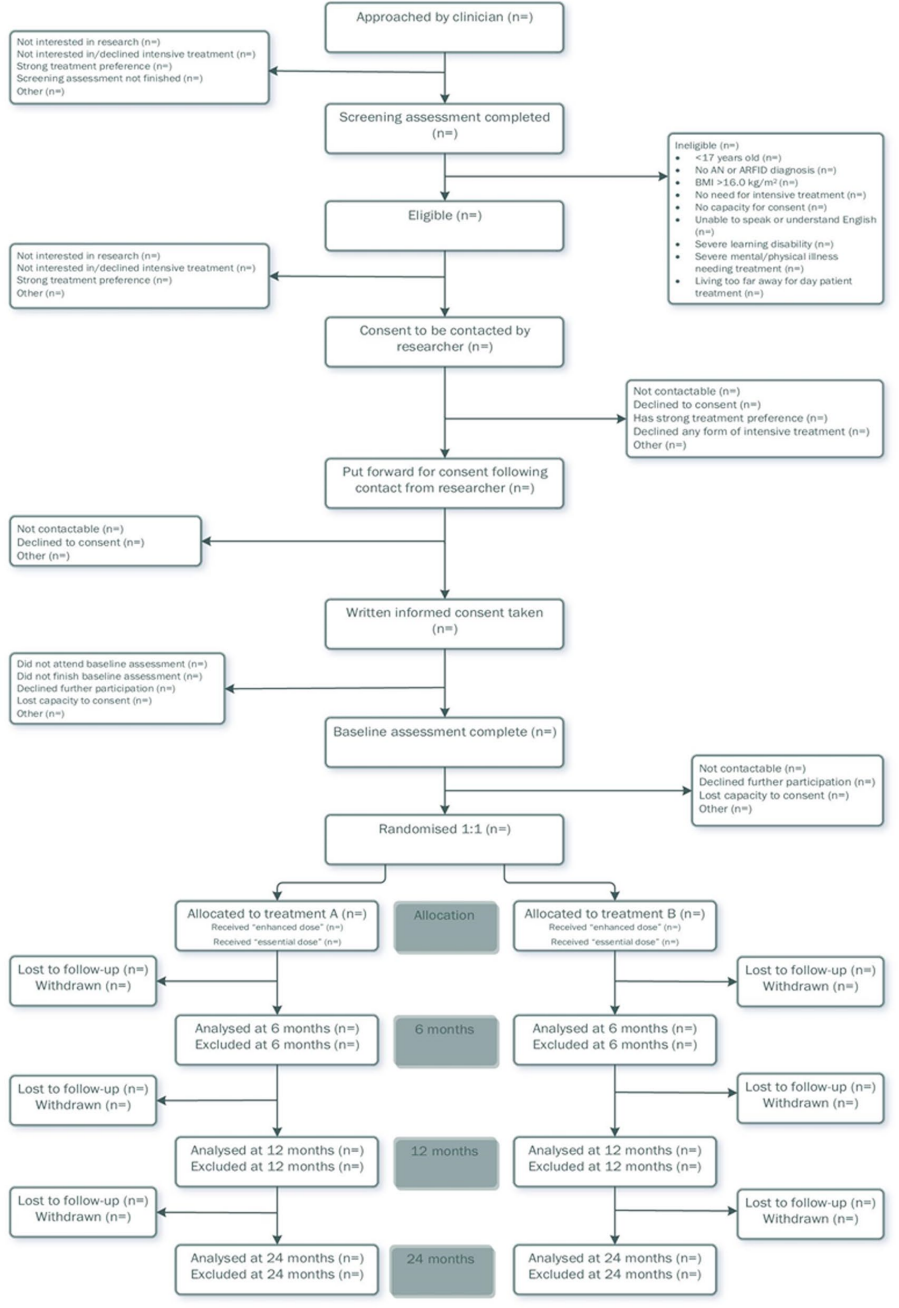
CONSORT diagram of the DAISIES trial

A trial-specific template CONSORT flowchart detailing the study procedure is presented in Fig. 1. Details about the schedule of enrolment, allocation and study assessments can be found in Table 1.

**Table 1:** Study schedule of enrolment, allocation and assessments

	Enrolment	Baseline (pre-randomisation)	Allocation	Monthly monitoring (randomisation–12 months post-randomisation)	6 months post-randomisation	12 months post-randomisation	24 months post-randomisation^**a**^
**Patient**							
**Enrolment:**							
Assessor checklist (eligibility screen)	**X**						
Intended treatment plan	**X**						
Informed consent	**X**						
Allocation			**X**				
**Assessment:**							
Demographics		**X**					
Body mass index (BMI)		**X**		**X**	**X**	**X**	**X**
Eating Disorder Examination interview (EDE) [58]		**X**			**X**	**X**	
Eating Disorder Examination Questionnaire (EDE-Q) [59]							**X**
Eating Disorder Examination Questionnaire Short (EDE-QS) [60]		**X**		**X**	**X**	**X**	
Autism Spectrum Quotient (AQ-10) [61]		**X**					
Depression, Anxiety and Stress Scales-Version 21 (DASS-21) [62]		**X**			**X**	**X**	**X**
Obsessive Compulsive Inventory-Revised (OCI-R) [63]		**X**			**X**	**X**	**X**
Clinical Impairment Assessment (CIA) [64]		**X**			**X**	**X**	**X**
Multidimensional Perceived Social Support Scale (MSPSS) [65]		**X**			**X**	**X**	**X**
Work and Social Adjustment Scale (WSAS) [66]		**X**			**X**	**X**	**X**
UCLA Loneliness Scale (Version 3) [67]		**X**			**X**	**X**	**X**
Motivational rulers (willingness and readiness to change)		**X**			**X**	**X**	**X**
Visual Analogue Scale (VAS) assessing treatment acceptability		**X**			**X**	**X**	**X**
Visual Analogue Scale (VAS) assessing treatment expectations		**X**					
Perceived Coercion Scale - Adapted (PCS) [68]		**X**			**X**		
Therapeutic Environment Scale (TESS) [69]				**X (at 3-months only)**			
Health-related Quality of Life (EQ-5D-5L) [70]		**X**			**X**	**X**	**X**
Adult Service Use Schedule (AD-SUS), designed for mental health populations [71, 72] and modified for AN.		**X**			**X**	**X**	**X**
Covid-19 diagnosis and symptom checklist		**X**			**X**	**X**	**X**
**Carer involvement (optional)**							
**Enrolment:**							
Informed consent	**X**						
**Assessment:**							
Demographics		**X**					
Eating Disorder Symptom Impact Scale (EDSIS) [73]		**X**			**X**	**X**	**X**
Depression, Anxiety and Stress Scales-Version 21 (DASS-21) [62]		**X**			**X**	**X**	**X**

^a^The 24-month post-randomisation assessment will be collected as part of a separate follow-up study. Approximately 60% of these will be collected during the current funding period and the remaining 40% will be subject to additional funding from the National Institute for Health and Care Research (NIHR)

#### Screening

Any patients deemed by their assessing or treating ED clinician to be in need of intensive treatment (in- or day patient treatment) will be invited to participate in the study. They will be approached by their assessing/treating clinician, informed that the study is taking place, and given a brief description of it. Whilst it is anticipated that most patients will be recruited from community/outpatient settings, some patients may be recruited from inpatient units, e.g. if they were originally admitted as an emergency and did not have capacity to consent to study participation prior to admission. For those interested, the clinician will complete a brief assessor checklist to screen for eligibility and assess the patient’s capacity. If eligible, the clinician will complete the decision tool (described above) and an intended treatment plan to assess whether the treatments pathways are being offered as planned. Clinicians will then ask the patient for consent to be contacted by the research team. The research team will provide further information, answer any questions and obtain written informed consent for randomisation and data collection from the patient. There is also an optional carer component designed to assess carer burden, and so, at this point patients will be asked for consent to involve their main carer within the research. If consent is given, the carer will be invited to participate, and informed consent for data collection will be obtained. Informed consent from both the patient and carer (if participating) will be obtained by a member of the research team before randomisation, either online (via Qualtrics), by post, or in person.

#### Baseline assessment

The patient baseline assessment will take place as soon as possible after informed consent has been given. It will consist of three parts which together take a maximum of 2 h 15 min to complete (weight and height measurement; approximately 60 min self-report questionnaires, and a clinical interview typically lasting no longer than 45 min). Participants will be sent a personal web link to access the self-report baseline questionnaires via Qualtrics. If preferred, questionnaires can be completed in person, or mailed out with a free postage stamp for return. The self-report questionnaires will usually be completed in the patient’s own time and will not have to be completed in a single session. The researchers will monitor questionnaire completion. Additionally, a phone, remote (via teleconferencing), or face-to-face appointment will be made to complete the Eating Disorder Examination (EDE) interview [58] and the interview-led section of the adapted Adult Service Use Schedule (AD-SUS; see below for further information) with a trained researcher. This interview will be audio-recorded (with patient consent) for quality assurance purposes. The researcher will record data from these interviews on paper or online (using Qualtrics) case report forms. Participants will be reimbursed £10 for the completion of the baseline assessment.

At this point, if consent has also been provided for carer participation, a short set of self-report questionnaires will be given to the carer for completion either online (via a unique Qualtrics link), by post or in person.

Once the patient and carer (if participating) have completed the baseline assessment, the patient will be randomly allocated to IP-TAU or the stepped care day patient approach, as described above. Once a participant has been randomised, no further carers can consent. The treating clinical team will then be informed of the allocated treatment arm by the designated unblinded member of the research team. The clinical team will inform the patient of their treatment allocation.

#### Patient symptom monitoring

Monthly patient symptom monitoring will take place to allow us to calculate remission and relapse rates. All patients will be asked to fill out the Eating Disorder Examination Questionnaire Short (EDE-QS, [60]) and self-report BMI to monitor ED symptoms on a monthly basis from the baseline assessment to 12 months post-randomisation. This will be completed via Qualtrics and accessed through a personal link sent via email 3 days prior to the due date. The researchers will monitor questionnaire completion. To ensure adherence to the symptom monitoring, if participants have not completed the questionnaire, they will be sent an email reminder at 3 days post-due date. Monthly BMI will also be obtained during this 12-month period, if possible, from the treating clinical team or the participant’s GP. As part of the symptom monitoring questionnaire at 3 months post-randomisation, the Therapeutic Environment Scale (TESS, [69]) will be administered. This measures various interpersonal processes in a therapeutic environment. Participants will be reimbursed £20 for completion of all monthly monitoring assessments.

#### Follow-up assessments

Follow-up assessments for both patients and carers will take place at 6 and 12 months post-randomisation. As mentioned previously, an additional 24-month follow-up study is planned but does not form part of the main trial evaluation. Patients will be contacted 2 weeks prior to the due date for these follow-up assessments to arrange an appointment (either by telephone, Microsoft Teams, or face-to-face) to complete the EDE interview and the interview-led section of the AD-SUS at 6 and 12 months post-randomisation. If participants and carers (if participating) have requested paper copies of the questionnaires, they will be mailed out at this point with a free postage stamp for return. If online completion of the questionnaires has been requested, the unique Qualtrics link to access the questionnaires will be emailed to the participant/carer 1 week prior to the due date. The researchers will monitor questionnaire completion. To ensure adherence to study assessment, if participants have not completed the questionnaires nor responded to our interview invitation by the assessment due date, participants will be contacted via telephone and, if required, will be sent further email reminders at 1 and 2 weeks post-due date. Participants will be reimbursed £10 for each completed follow-up assessment (6- and 12-month assessments only).

#### Process evaluation

A qualitative process evaluation will be conducted to investigate views on treatment and how it produced change from the perspective of patients, families and clinicians. Firstly, patients and carers will be approached following completion of the 6-month follow-up and invited to take part in a semi-structured interview. This will investigate positive and negative experiences of treatment, including its perceived short- and long-term effects. Participants will be recruited purposively across study sites to explore a range of opinions (e.g. according to gender, age, baseline ED symptoms, treatment motivation, expectations and experience). Secondly, semi-structured interviews will be conducted with inpatient, day patient and outpatient clinical staff over the course of the trial to investigate their experiences of managing patients, using the decision tool within the context of the two treatment arms, staff training, and their views on providing treatment to this patient group. Thirdly, study discussions about implementation, barriers and facilitators of the treatment approaches and learning events will be audio-recorded to provide further data on the perceived contextual factors at the patient, provider or system level that may contribute to variation in outcomes.

### Measures

Details of the study schedule of measurements and time points, including outcome measures to be collected as part of the additional 24-month follow-up study, can be found in Table 1. No biological samples will be collected in this study.

#### Primary outcome

The primary outcome is BMI (kg/m^2^) at 12 months post-randomisation.

#### Secondary outcomes

##### Clinical outcomes

- BMI (kg/m^2^) at 6 months post-randomisation.
- ED symptomology at 6 and 12 months post-randomisation, measured using the Eating Disorder Examination (EDE) interview [58].
- Comorbid symptomology at 6 and 12 months post-randomisation, measured using the Depression, Anxiety and Stress Scales-Version 21 (DASS-21, [62]) to assess mood, anxiety and stress symptoms and Obsessive Compulsive Inventory-Revised (OCI-R, [63]) to assess symptoms of obsessive compulsive disorder.

##### Psychosocial adjustment

- The Clinical Impairment Assessment (CIA, [64]) will assess psychosocial impairment associated with ED symptoms at 6 and 12 months post-randomisation.
- The Multidimensional Scale of Perceived Social Support (MSPSS, [65]) will assess the individual’s perceived social support at 6 and 12 months post-randomisation.
- The Work and Social Adjustment Scale (WSAS, [66]) will assess patients’ perceived functional impairments resulting from AN at 6 and 12 months post-randomisation.
- The UCLA Loneliness Scale (Version 3) [67] will assess patients’ subjective feelings of loneliness and social isolation at 6 and 12 months post-randomisation .

##### Motivation for change

- Motivational rulers (willingness and readiness to change) [53] will assess the importance of personal changes patients’ desire and evaluate their confidence about making those changes at 6 and 12 months post-randomisation.

##### Health-related quality of life

- The five-level version of the EuroQol measure of health-related quality of life (EQ-5D-5L, [70]), collected at 6 and 12 months post-randomisation, will be used to assess cost-effectiveness.

#### Additional measures

##### Baseline sample description

- All measures designated primary or secondary outcomes at a post-randomisation time point will also be measured at baseline.
- A purposely designed case record form will assess demographic characteristics of the participants, including gender, ethnicity, employment status, highest level of education, marital status, current living situation, current accommodation status and number of children/dependents, at baseline only.
- A purposely designed assessor checklist will record date of birth and assess clinical characteristics pertaining to the patient’s ED history, including age at ED onset, previous treatments received, and current treatment. This will be completed by the assessing clinician at baseline only.
- The Autism Spectrum Quotient (AQ-10, [61]) will assess autism spectrum traits at baseline only.

##### Remission and relapse rates

- BMI (kg/m^2^) on a monthly basis from baseline to 12 months post-randomisation.
- ED symptomology will be measured using the Eating Disorder Examination Questionnaire – short form (EDE-QS, [60]) on a monthly basis from baseline to 12 months post-randomisation.

##### Treatment expectations, acceptability, adherence and completion

- A visual analogue scale (VAS, [53]) will assess expectations of treatment effectiveness for both treatment approaches at baseline only.
- A visual analogue scale (VAS, [53]) will assess experienced treatment acceptability of their allocated treatment at 6 and 12 months post-randomisation, adjusting for expected treatment acceptability at baseline.
- Intended treatment plan will estimate patient’s treatment need at baseline only.
- Admission and discharge dates to ED inpatient and day patient services.
- Number of inpatient days or number of day treatment days (including proportion of remote versus in person days) attended per week.

##### Economic measures

- Adult Service Use Schedule (AD-SUS), designed for mental health populations [71, 72] and modified for AN, at baseline, 6 and 12 months post-randomisation. This will include an assessment of all hospital and community health and social care services plus prescribed medications.
- Hospital Episode Statistics (HES) data from NHS Digital (for participants from England) or Information Services Division (ISD; for participants from Scotland). Data requested will include the number of admission days to Accident & Emergency, specialist ED units and general psychiatric inpatient wards in the year prior to participation in the study, and for 2 years post-randomisation.

##### Covid-19-related variables

- A Covid-19 diagnosis and symptom checklist will assess whether participants have been diagnosed with or had suspected Covid-19 and the symptoms they experienced [74], and whether they have received a Covid-19 vaccination at baseline, 6 and 12 months post-randomisation.

##### Carer burden

- A purposely designed record form will assess demographic characteristics of the carers (if participating) including age, gender, ethnicity, employment status, the nature of their relationship to the study participant and whether they live with the participant at baseline only.
- Depression, Anxiety and Stress Scales-Version 21 (DASS-21, [62]) will assess mood, anxiety and stress symptoms at baseline, 6 and 12 months post-randomisation.
- Eating Disorders Symptom Impact Scale (EDSIS, [73]) will assess the carer’s perceptions of ED-specific burden at baseline, 6 and 12 months post-randomisation.

##### Collected as part of the trial for future secondary analyses (i.e. not presented in or analysed as part of the primary publication)

Treatment experience includes the following:

- The Perceived Coercion Scale (PCS, [68]) will assess the influence, freedom, control and choice regarding the admission process at baseline and 6 months post-randomisation.
- The Therapeutic Environment Scale (TESS, [69]) will assess the occurrence of various interpersonal processes in a therapeutic environment at 3 months post-randomisation (included as part of the third symptom monitoring questionnaire).

### Data management

The trial researchers will create a Data Management Plan to document how data will be collected, handled, stored and checked. This will be made available to the trial analysts. Data collected (from paper case report forms and downloaded source data records from Qualtrics) will be entered onto electronic case report forms within the Elsevier InferMed MACRO Version 4 data capture system hosted by Kings Clinical Trials Unit. Two linked online data entry systems will be created to maintain blinding; the first will be used by all approved researchers to enter outcome and additional measure data, and the second will be used by the allocated unblinded researcher to enter trial outcome data with the potential to cause unblinding (including the intended treatment plan, treatment attendance, the Covid-19 checklist, the AD-SUS and adverse events).

Identifying information from participant data will be removed, stored separately and replaced with a unique numeric code, which will be used to identify their data. The master list of names which correspond to participants’ numeric identification codes will be stored electronically and will be password-protected. This information will only be accessible to key researchers involved in the study. All personal and pseudo-anonymised data will either be stored in securely locked filing cabinets at the Section of Eating Disorders, Institute of Psychiatry, Psychology & Neuroscience, King’s College London, or on university servers (on university desktop and laptop computers) and university-approved online storage systems/databases, all of which are password-protected and encrypted. All investigators will have access to the final trial data set without any limits.

### Data monitoring

A Data Monitoring and Ethics Committee has been established due to the risk associated with severe AN. This committee is independent of the study organisers. The Data Monitoring and Ethics Committee will meet on a yearly basis throughout the trial and undertake interim review of the trial’s progress, in the strictest confidence, by reviewing and monitoring trial data, including recruitment figures, data quality, protocol compliance and treatment harm (i.e. adverse events). They will directly report any recommendations or feedback to the Trial Steering Committee who provide overall supervision on behalf of the Project Sponsor and Project Funder and ensure the study is conducted to the rigorous standards set out in the Department of Health’s Research Governance Framework for Health and Social Care and the Guidelines for Good Clinical Practice. The Chair of the Data Monitoring and Ethics Committee is Dr Jacinta Tan and the members are Dr Jane Morris and Dr Dennis Görlich. Terms of Reference for the Data Monitoring and Ethics Committee are available on request from the study research team.

#### Premature termination of the trial

No interim analyses will be performed to inform premature termination of the trial. The trial may be prematurely discontinued by the Sponsor or Principal Investigator on the basis of new safety information or for other reasons given by the Data Monitoring and Ethics Committee/Trial Steering Committee, regulatory authority or ethics committee concerned. The trial may also be prematurely discontinued due to lack of recruitment within the internal pilot study. Specifically, we have a stopping guideline in place for if <50% of the desired internal pilot sample size (*n* = 62) is recruited. If the study is prematurely discontinued, active participants will be informed, and no further participant data will be collected.

#### Harms

Adverse events will be spontaneously reported by site investigators to the trial’s research team. Data on adverse events will be collected throughout the trial, and all adverse events will be recorded in electronic case report forms within the trial’s online data entry system. Specifically, we will record the following information: type of adverse event (e.g. physical – weight loss); full details and case description; approximate date of initial occurrence; relationship to the general study procedures or study care pathway (e.g. related or unrelated); whether the event would be considered expected or unexpected; and whether the event is classed as serious. If deemed serious, the adverse event will be escalated to the Principal Investigator who will determine if the event was related to the study and unexpected. If this is the case, the adverse event will be reported to the Sponsor. No follow-up care for adverse events will be given as the intervention does not involve the use of drugs. All adverse events will be reported to the relevant monitoring committees and will be presented as part of the final trial analysis. There is no obvious risk for participants, and we do not expect any adverse events.

### Data analysis

A detailed statistical analysis plan will be developed by the trial statisticians whilst maintaining full blind and agreed with the research team and Data Monitoring and Ethics Committee before the end of the internal pilot. Briefly, all formal comparisons of the two care pathways will follow the intention-to-treat principle. For continuous clinical outcomes variables, such as BMI, linear mixed models will be fitted to the outcome measures at 6 and 12 months to estimate the trial arm differences. The fixed part of these models will contain the explanatory variables of interest (trial arm, time and an interaction term) and also baseline values of outcome variables and randomisation stratifiers, as these variables are known to explain variability in outcome. To account for correlation between the repeated measures at 6 and 12 months, a subject-varying random intercept will be included. For non-continuous outcomes, relevant generalised linear mixed models will be fitted. Results are valid under a missing at random (MAR) missing data generating mechanism that allows earlier outcome values to predict missingness of later ones. In the presence of missing data, should we find that the assumptions underlying the mixed modelling approach are not realistic, then an alternative, more flexible approach such as multiple imputation will be used instead.

To judge non-inferiority of the stepped care approach in terms of the primary outcome (BMI at 12 months), a two-sided 95% confidence interval of the trial arm difference in this clinical measure will be generated and this interval compared with the non-inferiority threshold (0.75 kg/m^2^ decrease in BMI). The threshold defines a region for the trial arm difference in BMI in which the stepped care day patient approach would be considered non-inferior to IP-TAU. To confirm the non-inferiority hypothesis, the whole 95% confidence interval would need to lie within this region. Supplementary subgroup analyses will estimate care pathway differences in treatment-completer populations (defined using the minimal and full treatment completion criteria described previously) to investigate the impact of any treatment non-completion on the non-inferiority assessment. In this context, methods from the causal inference field, e.g. inverse probability weighting, will be used to adjust for variables driving both treatment completion and primary BMI outcome [75]. No other subgroup analyses are planned.

#### Economic evaluation

The economic evaluation will take the NHS/personal social services perspective preferred by the National Institute for Health and Care Excellence (NICE) and shown in previous AN studies to constitute over 90% of total societal costs. Service use data will be collected using the Adult Service Use Schedule (AD-SUS), designed for use in adult populations with mental health conditions [71, 72] and modified for AN on the basis of relevant literature and clinical expertise. The AD-SUS is used to assess the number and length of contacts with various health and social care services and professionals, plus prescribed medications, over a period of time appropriate for each individual study. The DAISIES AD-SUS will be used at baseline to collect use of services in the previous 6 months and at the 6- and 12-month follow-ups to collect use of services since the previous interview. In this way, the full period from baseline to the 12-month follow-up will be covered. The AD-SUS is commonly administered in interview format. However, to avoid unblinding the research assessors, use of hospital services (inpatient, day patient, outpatient and Accident & Emergency) will be reported by participants using a self-report version of the AD-SUS. All remaining service use (i.e. community services and prescribed medications) will be collected in interview. This is to improve recall accuracy, whilst ensuring that the researchers remain blinded. In addition, the collection of data on use of hospital services from clinical records at each site will be piloted at baseline and, if successful, implemented at each follow-up. The self-report data will be secondary to the clinical records data and will be used where clinical data cannot be provided. Nationally applicable unit costs will be applied to all services, including the inpatient and day patient-based interventions [76, 77]. To supplement these data, Hospital Episode Statistics (HES) from NHS Digital (for participants from England) and Information Services Division (ISD; for participants from Scotland) will also be requested and incorporated if data is received in a timely fashion.

Cost-effectiveness will be explored at the 12-month follow-up in terms of QALYs measured using the EQ-5D-5L [70], with a sensitivity analysis using the primary clinical measure of outcome (BMI), given the limited evidence available for the validity of the EQ-5D-5L in ED populations. QALYs will be calculated by applying appropriate utility weights to health states [78] and using the total area under the curve approach with linear interpolation between assessment points [79].

Mean differences in costs and 95% confidence intervals will be obtained by nonparametric bootstrap regressions to account for the non-normal distribution often found in economic data. Cost-effectiveness analyses will be undertaken irrespective of whether non-inferiority is demonstrated, since exploration of the joint distribution of costs and effects is recommended to represent uncertainty [80] and to help interpret the economic results [81]. Incremental cost-effectiveness ratios (ICERs) will be calculated if higher costs and better outcomes are found in either the stepped care day patient approach or IP-TAU group. Uncertainty around cost-effectiveness estimates will be explored using cost-effectiveness planes and cost-effectiveness acceptability curves (CEACs) based on the net-benefit approach [82]. All economic analyses will be adjusted in line with the clinical approach (baseline values of the variables of interest and randomisation stratifiers). Missing economic cost and outcome data will be imputed using multiple imputation methods by using chained equations, under the assumption that these data were missing at random [83].

#### Qualitative analysis

Qualitative data will be analysed using the Framework Approach [84] to facilitate analysis within and between individual cases and groups of participants. The thematic framework will draw on a priori issues around perceived mechanisms of impact, implementation and context, but be responsive to emergent and analytical themes. Once applied to individual transcripts, data will be charted to map and interpret the data set as a whole. Qualitative process data will be analysed prior to the outcome data, but any insights will not be communicated to the wider team until the RCT outcomes are known.

### Patient and public involvement

The initial study proposal was discussed with 12 patients with severe AN who had experience of day treatment, inpatient treatment or both. Patients strongly endorsed the design of the present study, in particular the stepped care aspect, as this was felt to offer individually tailored care. Measures of treatment motivation, perceived coercion, social support and loneliness were added to the study, based on patient feedback. Two patients and a carer reviewed and commented on the final proposal. Three people (two patients, one carer) will become patient and public involvement (PPI) representatives. Additionally, qualitative semi-structured interviews with PPI representatives (who have received intensive treatments for severe anorexia nervosa or a related disorder and carers of such patients) will be conducted to inform recruitment strategies and study implementation.

## Ethics and dissemination

### Ethics and safety aspects

Ethical approval was received from Wales Research Ethics Committee 5 (Reference: 20/WA/0072), and all recruiting sites will obtain individual approvals through their local Research and Development departments. Written informed consent to participate in the study will be obtained from all participants by the research team. It will be made clear to participants that they can withdraw from the study at any time without justification. In addition, participants will be informed that if they withdraw from the study intervention only, the research team will continue to make efforts to obtain follow-up data, unless requested otherwise.

The Trial Management Group will be responsible for the day-to-day management of the trial. The trial will also be overseen by two independent committees, the Data Monitoring and Ethics Committee and the Trial Steering Committee (as described above). All committees will be responsible for ensuring the trial is run in compliance with the trial protocol, the Declaration of Helsinki, the Principles of Good Clinical Practice (ICH-E6 guideline) and in accordance with all applicable regulatory requirements. Any substantial protocol amendments will be first discussed with the Sponsor and the National Institute for Health and Care Research (NIHR), before submitting an amendment to the Research Ethics Committee. Standard King’s College London insurance and NHS indemnity arrangements apply to this study.

To ensure patient safety and inform treatment step-up/step-down, whilst in treatment, weekly decision tools will be conducted by the clinical team, as described above. The decision tool will comprehensively assess medical risk, using objective indicators of nutritional status, cardiovascular function, laboratory parameters, other physical risk indications and psychosocial risk. A risk reference committee will also be established for discussion or advice on any risk concerns.

Patients enrolled into the study are covered by indemnity for harm arising from the conduct of the study through the standard NHS indemnity arrangements. King’s College London has insurance to cover for harm arising from the management and design of the protocol.

### Dissemination policy

The results of this study will be reported and disseminated at national and international conferences and in peer-reviewed open-access scientific journals. We will also disseminate findings through relevant websites and social media. Finally, we will work closely with NHS England to implement findings.

An Authorship Policy will be developed by the Trial Management Group to determine the criteria that are required to be met for authorship. For each publication, authorship will be decided by all of the people involved in conducting the study with reference to the Authorship Policy. No professional writers will be used.

## Discussion

Inpatient and/or day patient treatment are recommended for severe AN by international guidelines [10, 85]. However, there is limited previous research regarding clinical outcomes and the cost-effectiveness of intensive treatment approaches for severe AN [19, 21, 26]. The DAISIES trial is the first RCT to compare inpatient care to a stepped care day patient approach for adults with severe AN. This is a complex trial which will take participating services out of their ‘comfort zone’ and require them to substantially change their clinical practice and service protocols (e.g. by stepping patients down from inpatient to day patient treatment early and accepting more unwell patients into day treatment). It is for these reasons that the internal pilot and qualitative aspects of the trial will be of great importance to the ongoing day-to-day implementation of trial procedures, e.g. developing effective recruitment strategies. In addition, we intend to start the trial during the Covid-19 pandemic, with services running at a reduced capacity, which poses additional challenges. Despite these challenges, it is hoped that the results of this study will provide a rigorous evaluation of two intensive treatment approaches which will inform future NICE and international treatment guidelines and service provision.

## Trial status

Protocol version 6, 15 June 2021.

Participant recruitment and data collection for this trial commenced in November 2020 and is expected to run until April 2022.

Sponsor: King’s College London (KCL) is the lead sponsor and South London and Maudsley NHS Foundation Trust is the co-sponsor for this study. Sponsor contact details are as follows: Professor Reza Razavi, Room 5.31, James Clerk Maxwell Building, 57 Waterloo Road, London, SE1 8WA, United Kingdom. The sponsors will act as the data controllers for this study.

## Data Availability

The trial protocol will be available upon request from the Principal Investigator and the full study report, anonymised data set and statistical code will be made available 24 months after the final data set has been obtained.

## Abbreviations

AD-SUS: Adult Service Use Schedule
AN: Anorexia nervosa
AQ-10: Autism Spectrum Quotient
ARFID: Avoidant restrictive food intake disorder
BMI: Body mass index
CEAC: Cost-effectiveness acceptability curves
CIA: Clinical Impairment Assessment
DASS-21: Depression, Anxiety and Stress Scales-Version 21
DSM: Diagnostic and Statistical Manual of Mental Disorders
ED: Eating disorder
EDE: Eating Disorder Examination
EDE-Q: Eating Disorder Examination Questionnaire
EDE-QS: Eating Disorder Examination Questionnaire Short
EDSIS: Eating Disorder Symptom Impact Scale
HES: Hospital Episode Statistics
ICER: Incremental cost-effectiveness ratios
IP-TAU: Inpatient treatment as usual
ISD: Information Services Division
KCTU: King’s Clinical Trials Unit
MAR: Missing at random
MSPSS: Multidimensional Perceived Social Support Scale
NHS: National Health Service
NICE: National Institute for Health and Care Excellence
NIHR: National Institute for Health and Care Research
OCI-R: Obsessive Compulsive Inventory-Revised
PCS: Perceived Coercion Scale
PPI: Patient and public involvement
QALY: Quality-adjusted life-years
RCT: Randomised controlled trial
TESS: Therapeutic Environment Scale
VAS: Visual analogue scale
WSAS: Work and Social Adjustment Scale
